# Measures of clinical malaria in field trials of interventions against *Plasmodium falciparum*

**DOI:** 10.1186/1475-2875-6-53

**Published:** 2007-05-02

**Authors:** Thomas A Smith

**Affiliations:** 1 Department of Public Health & Epidemiology, Swiss Tropical Institute, Socinstrasse 57, Postfach CH-4002, Basel, Switzerland

## Abstract

**Background:**

Standard methods for defining clinical malaria in intervention trials in endemic areas do not guarantee that efficacy estimates will be unbiased, and do not indicate whether the intervention has its effect by modifying the force of infection, the parasite density, or the risk of pathology at given parasite density.

**Methods:**

Three different sets, each of 500 Phase IIb or III malaria vaccine trials were simulated corresponding to each of a pre-erythrocytic, blood stage, and anti-disease vaccine, each in a population with 80% prevalence of patent malaria infection. Simulations considered only the primary effects of vaccination in a homogeneous trial population. The relationships between morbidity and parasite density and the performance of different case definitions for clinical malaria were analysed using conventional likelihood ratio tests to compare incidence of episodes defined using parasite density cut-offs. Bayesian latent class models were used to compare the overall frequencies of clinical malaria episodes in analyses that did not use diagnostic cut-offs.

**Results:**

The different simulated interventions led to different relationships between clinical symptoms and parasite densities. Consequently, the operating characteristics of parasitaemia cut-offs in general differ between vaccine and placebo arms of the simulated trials, leading to different patterns of bias in efficacy estimates depending on the type of intervention effect. Efficacy was underestimated when low parasitaemia cut-offs were used but the efficacy of an asexual blood stage vaccine was overestimated when a high parasitaemia cut-off was used. The power of a trial may be maximal using case definitions that are associated with substantial bias in efficacy.

**Conclusion:**

Secondary analyses of the data of malaria intervention trials should consider the relationship between clinical symptoms and parasite density, and attempt to estimate overall numbers of clinical episodes and the degree of bias of the primary efficacy measure. Such analyses would help to clarify whether the effect of an intervention corresponds to that anticipated on the basis of the parasite stage that is targeted, and would highlight whether the primary measure of efficacy results from unexpected behaviour in the parasitological and clinical data used to estimate it.

## Background

In endemic settings malaria usually presents with rather non-specific symptoms, such as fever, and not all sick individuals with malaria parasites are really suffering from clinical malaria. This is because most of the population may be infected with *Plasmodium falciparum *parasites, without these causing any acute illness. It follows that the presence of parasites in a sick person does not necessarily mean that malaria is the cause of the illness. In field trials of novel interventions, estimates of efficacy need to be made using case definitions with high specificity. Otherwise efficacy will be underestimated.

The greater the parasite density in the blood the more reasonable it is to assume that an illness is caused by malaria, so a case definition for clinical malaria for use in a trial can be obtained by defining a parasite density cut-off specific for the surveillance mechanism of choice (local health centre, hospital, active case detection). The sensitivity and specificity of different parasite density cut-offs can be obtained by modeling the excess risk of fever as a function of parasite density [1,2], where the comparator is the risk in aparasitaemic individuals. This has been used in a number of trials to decide upon an appropriate cut-off [3-5]. Ideally this analysis is carried out in the same population (and age groups) as the vaccine trial and using the same morbidity surveillance system, since relationships between morbidity and infection depend on age and immune status [6-8].

This approach has been endorsed by the WHO Study Group on Measures of Malaria Vaccine Efficacy for obtaining case definitions for use in pivotal trials of malaria vaccine [9]. Efficacy estimates based on this algorithm can be easily obtained using standard software and are appropriate for defining the primary outcome for trials aiming to achieve registration.

Applying such an algorithm though does not guarantee that efficacy estimates will be unbiased, and does not provide an interpretation of how a vaccine is acting. As secondary objectives of malaria vaccine trials, investigators should be interested also in drawing inferences about whether the vaccine acts in accordance with its design and how it interacts with natural immunity. This paper uses simulations of trials to consider the theoretical performance of this method for different kinds of vaccines, and suggests a range of additional exploratory analyses that can be carried out in order to better understand vaccine action. The simulations consider the likely effects of different kinds of vaccines but the same approach is applicable to the analysis of the clinical impact of any effective intervention against malaria.

## Methods

### Simulations

**Figure 1:**
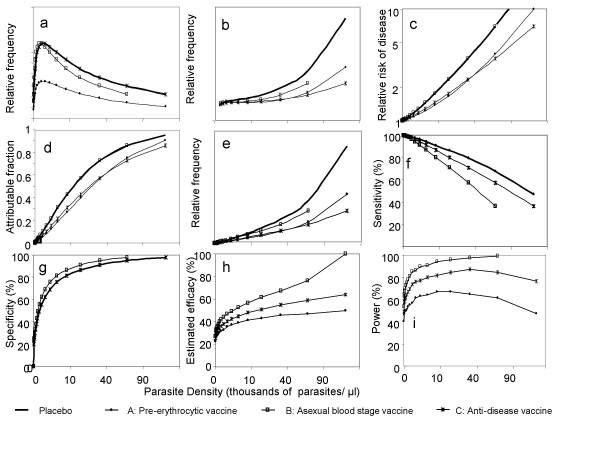
**Results of simulated vaccine trials**. Description of each sub-figure is given in Table 2.

Phase IIb or III malaria vaccine trials were simulated assuming the study population to comprise children with an 80% prevalence of *P. falciparum *malaria, a distribution of parasite densities in the population, *θ*_*c*_(*x*), (in the absence of vaccine or in a placebo group) as shown in the thick line Figure 1a. The simulated frequency of disease in the placebo group at different parasite densities (relative to the risk in aparasitaemic individuals) is shown in the thick line in Figure 1b, with 45.5% of the clinical episodes attributable to malaria (Table 1). Three different hypothetical vaccine effects were simulated (Table 1):

**Table 1:** Properties of hypothetical vaccines

			**Vaccine effect**		
		**Placebo**	**A: Reduction in force of infection by 50%**	**B: Reduction in parasite densities by 50%**	**C: Multi-plication of pyrogenic threshold by 2**

Prevalence of patent infection		80%	40%	77%	80%
Relative incidence of disease	All episodes	100.0	77.2	74.0	74.0
	Malaria	45.5	22.8	19.6	19.6
	Non malaria	54.5	54.5	54.5	54.5
True efficacy in preventing clinical malaria episodes		-	50%	57%	57%
True efficacy in preventing any disease episode		-	22.8%	26.0%	26.0%

Vaccine A: (Pre-erythrocytic vaccine). This vaccine reduces the force of infection by 50%. For simplicity, this is assumed to be reflected in a 50% reduction in the proportion of individuals who are infected across the whole range of parasite densities, and a random sample of 50% of the clinical malaria episodes are assumed to be averted.

Vaccine B: (Asexual blood stage vaccine). This vaccine reduces parasite densities by 50%, but does not affect the number of individuals who are infected, or the parasite densities at which they become ill.

Vaccine C: (Anti-disease vaccine). This vaccine is assumed to have no effect on parasite densities but to lead to an increase in the parasite density at which clinical malaria occurs.

For each of these models of vaccination 500 simulated trials were run. Each simulated trial had a standard design with (i) an equal number of placebo and vaccine recipients; (ii) a total of 100 parasitological slides in each arm used to estimate the effect on the parasite density distribution in the population, and (iii) a standardised clinical surveillance to detect fever cases, with a total of 100 cases expected in unvaccinated individuals.

Datasets were constructed from these simulations using 19 categories of parasite density. The community parasitaemia data were generated by defining multinomial probabilities for each of the 19 categories based on the data of Figure 1a and randomly drawing a sample of 100 parasite densities from this distribution. The relative frequencies in Figure 1b were used to define the expected number of clinical cases for each of these 19 categories, on the assumption that the expected total number of clinical cases in the placebo arm was 100. The simulated number of cases in each category was then drawn from a Poisson distribution.

### Analyses of effects of simulated vaccines

The effects of the different hypothetical vaccines were summarized both by considering the theoretical effects on *θ*_*c*_(*x*), the parasite density distribution in the community (Figure 1a), and on *θ*_*s*_(*x*) the density in sick individuals (Figure 1b). The latter arises as a mixture:

*θ*_*s*_(*x*) = *λθ*_*m*_(*x*) + (1-*λ*)*θ*_*c*_(*x*)

where *θ*_*m*_(*x*) is the parasite density distribution in patients with clinical malaria and *λ *is the malaria attributable fraction. *θ*_*s*_(0), *θ*_*c*_(0) are then the frequencies of the uninfected (aparasitaemic) classes, and the relative risk of given parasite density *x *in sick individuals relative to controls (Figure 1c) is given by θs(x)θc(0)θc(x)θs(0)
 MathType@MTEF@5@5@+=feaafiart1ev1aqatCvAUfKttLearuWrP9MDH5MBPbIqV92AaeXatLxBI9gBaebbnrfifHhDYfgasaacH8akY=wiFfYdH8Gipec8Eeeu0xXdbba9frFj0=OqFfea0dXdd9vqai=hGuQ8kuc9pgc9s8qqaq=dirpe0xb9q8qiLsFr0=vr0=vr0dc8meaabaqaciaacaGaaeqabaqabeGadaaakeaadaWcaaqaaGGaciab=H7aXnaaBaaaleaacqWGZbWCaeqaaOGaeiikaGIaemiEaGNaeiykaKIae8hUde3aaSbaaSqaaiabdogaJbqabaGccqGGOaakcqaIWaamcqGGPaqkaeaacqWF4oqCdaWgaaWcbaGaem4yamgabeaakiabcIcaOiabdIha4jabcMcaPiab=H7aXnaaBaaaleaacqWGZbWCaeqaaOGaeiikaGIaeGimaaJaeiykaKcaaaaa@4577@. At each parasite density *x*, *λ*(*x*) is the malaria attributable proportion of clinical cases at *x*, equal to:

λ(x)=1−θc(x)θs(0)θs(x)θc(0)
 MathType@MTEF@5@5@+=feaafiart1ev1aqatCvAUfKttLearuWrP9MDH5MBPbIqV92AaeXatLxBI9gBaebbnrfifHhDYfgasaacH8akY=wiFfYdH8Gipec8Eeeu0xXdbba9frFj0=OqFfea0dXdd9vqai=hGuQ8kuc9pgc9s8qqaq=dirpe0xb9q8qiLsFr0=vr0=vr0dc8meaabaqaciaacaGaaeqabaqabeGadaaakeaaiiGacqWF7oaBcqGGOaakcqWG4baEcqGGPaqkcqGH9aqpcqaIXaqmcqGHsisldaWcaaqaaiab=H7aXnaaBaaaleaacqWGJbWyaeqaaOGaeiikaGIaemiEaGNaeiykaKIae8hUde3aaSbaaSqaaiabdohaZbqabaGccqGGOaakcqaIWaamcqGGPaqkaeaacqWF4oqCdaWgaaWcbaGaem4CamhabeaakiabcIcaOiabdIha4jabcMcaPiab=H7aXnaaBaaaleaacqWGJbWyaeqaaOGaeiikaGIaeGimaaJaeiykaKcaaaaa@4D34@

Figure 1d gives values for this quantity for each of the simulated populations and Figure 1e gives the distributions of *θ*_*m*_(*x*). The sensitivity of a cut-off *X *is then given by:

Pr⁡(x≥X|malaria episode)=∫X∞θm(x)dx
MathType@MTEF@5@5@+=feaafiart1ev1aqatCvAUfKttLearuWrP9MDH5MBPbIqV92AaeXatLxBI9gBaebbnrfifHhDYfgasaacH8akY=wiFfYdH8Gipec8Eeeu0xXdbba9frFj0=OqFfea0dXdd9vqai=hGuQ8kuc9pgc9s8qqaq=dirpe0xb9q8qiLsFr0=vr0=vr0dc8meaabaqaciaacaGaaeqabaqabeGadaaakeaacyGGqbaucqGGYbGCcqGGOaakcqWG4baEcqGHLjYScqWGybawcqGG8baFcqqGTbqBcqqGHbqycqqGSbaBcqqGHbqycqqGYbGCcqqGPbqAcqqGHbqycqqGGaaicqqGLbqzcqqGWbaCcqqGPbqAcqqGZbWCcqqGVbWBcqqGKbazcqqGLbqzcqGGPaqkcqGH9aqpdaWdXbqaaGGaciab=H7aXnaaBaaaleaacqWGTbqBaeqaaOGaeiikaGIaemiEaGNaeiykaKIaemizaqMaemiEaGhaleaacqWGybawaeaacqGHEisPa0Gaey4kIipaaaa@59FA@

(Figure 1f) and the specificity (Figure 1g) by:

Pr⁡(malaria episode | x≥X)=ψ(X)=∫X∞λ(x)θs(x)dx∫X∞θs(x)dx
MathType@MTEF@5@5@+=feaafiart1ev1aqatCvAUfKttLearuWrP9MDH5MBPbIqV92AaeXatLxBI9gBaebbnrfifHhDYfgasaacH8akY=wiFfYdH8Gipec8Eeeu0xXdbba9frFj0=OqFfea0dXdd9vqai=hGuQ8kuc9pgc9s8qqaq=dirpe0xb9q8qiLsFr0=vr0=vr0dc8meaabaqaciaacaGaaeqabaqabeGadaaakeaacyGGqbaucqGGYbGCcqGGOaakcqqGTbqBcqqGHbqycqqGSbaBcqqGHbqycqqGYbGCcqqGPbqAcqqGHbqycqqGGaaicqqGLbqzcqqGWbaCcqqGPbqAcqqGZbWCcqqGVbWBcqqGKbazcqqGLbqzcqqGGaaicqqG8baFcqqGGaaicqWG4baEcqGHLjYScqWGybawcqGGPaqkcqGH9aqpiiGacqWFipqEcqGGOaakcqWGybawcqGGPaqkcqGH9aqpdaWcaaqaamaapehabaGae83UdWMaeiikaGIaemiEaGNaeiykaKIae8hUde3aaSbaaSqaaiabdohaZbqabaGccqGGOaakcqWG4baEcqGGPaqkcqWGKbazcqWG4baEaSqaaiabdIfaybqaaiabg6HiLcqdcqGHRiI8aaGcbaWaa8qCaeaacqWF4oqCdaWgaaWcbaGaem4CamhabeaakiabcIcaOiabdIha4jabcMcaPiabdsgaKjabdIha4bWcbaGaemiwaGfabaGaeyOhIukaniabgUIiYdaaaaaa@749F@

To compare the efficacy in averting clinical episodes that might be estimated in the simulated trials two distinct analyses of all 500 sets of simulated trials were carried out:

1. In the first analysis, efficacy was estimated as the median of the sample of 1-I_V_(X)/I_P_(X) in the 500 simulated trials, where where I_V_(X) is the number of simulated cases in vaccinees, with parasite density>X; and I_P_(X) is the number of simulated cases in the placebo arm (Figure 1h). Each of the category boundaries (X) used to sample the data was used in turn as a cut-off, and the average of the resulting efficacy estimates plotted against the cut-off (Figure 1h).

The power of these analyses was then estimated by determining the proportion of the sample of 500 simulated trials that gave significant efficacy as assessed using using binomial likelihood ratio tests (two sided) of the null hypothesis I_V_(X) = I_P_(X) (significance level α = 0.05).

2. The second analysis sought to estimate the number of clinical malaria cases in each arm of each of the 500 simulated trials, by assigning a probability to each fever case, as a function of parasite density, rather than by using a dichotomous classification. These probabilities were estimated using a latent class model [10,11] as previously described. This algorithm, implemented separately for vaccine and placebo, involved comparing the parasite density distributions in the simulated cases with those in the community samples drawn from *θ*_*c*_(*x*).

## Results

The parasite density distributions in the community (Figure 1a) vary as straightforward consequences of the primary effects of vaccination. The pre-erythrocytic vaccine (A) halves the number of slide positive individuals in each category (a simplification of what we expect in a field study, where superinfection may occur); the effect of the simulated asexual blood stage vaccine (B) is more complicated, as it disproportionately reduces the frequency of high parasite densities, and slightly increases the frequency of very low parasite densities by shifting each individual to a lower density (Figure 1a). One consequence of this is that the highest density class is not represented among individuals who receive vaccine B, since any individual who would have been in this density class is now in the second highest class. The simulated anti-disease vaccine (C) has no effect on the parasite density distribution in the community.

The distributions of parasite densities in the clinical cases differ among the three vaccines (Figure 1b). For all the vaccines there is a background incidence of non-malaria disease, which is assumed to occur independently of the parasite density (left hand side of figure 1b), corresponding to non-malaria illness and is the same in all groups. For all three vaccines fewer cases are expected at each positive value of the density distribution than occur in the placebo group.

These differences in parasite density distributions lead in turn to different relationships between incidence of disease and the community parasite density distribution, depending on the action of the vaccine (Figure 1c), and hence to different curves for the relationship between the attributable fraction, the frequencies of clinical cases with different densities, and the operating characteristics of case definitions (Figure 1defg, Table 2). The relative risk of a given parasite density among cases, relative to the risk in controls, is the same for the anti-blood stage vaccine B as for the placebo arm (because the risk of disease, conditional on the parasite density, is the same in both arms, and the number of disease cases with no parasites is unchanged by the vaccine). For vaccine A, the proportion of cases at any given positive density is lower than in the corresponding proportion of cases in placebo recipients, because more of the cases with non-malaria etiology are now aparasitaemic, so the relative risk of a given parasite density among cases relative to controls is lower than in placebo (Figure 1c). For vaccine C the curve in Figure 1c is also lower than that for placebo, but this is because there are fewer parasitaemic cases- there is no change in the number of aparasitaemic ones.

**Table 2:** Results of simulated vaccine trials

	**Vaccine effect**		
	**A: Reduction in force of infection by 50%**	**B: Reduction in parasite densities by 50%**	**C: Multiplication of pyrogenic threshold by 2**

Figure 1a: Distribution of parasite densities in the community (from which simulated datasets are sampled)	Frequency is halved at each density above zero. Frequency of zero parasite density increases to compensate.	Frequency of low parasite densities increases; frequency of high parasite densities decreases. Frequency of zero parasite density unchanged.	Same as placebo
Figure 1b: Distribution of parasite densities in all disease cases 1b) (from which simulated datasets are sampled)	Frequency relative to that in placebo decreases with increasing parasite density.	Frequency relative to that in placebo decreases with increasing parasite density	Frequency relative to that in placebo decreases with increasing parasite density
Figure 1c: Relative risk of given parasite density in disease cases relative to controls	At any given density, reduced relative to placebo	Same as placebo	At any given density, reduced relative to placebo
Figure 1d: Attributable fraction of cases by parasite density (Figure 1d)	At any given density, reduced relative to placebo	Same as placebo	At any given density, reduced relative to placebo
Figure 1e: Distribution of parasite densities in clinical malaria cases	Frequency of high parasite densities lower than in placebo	Frequency of high parasite densities lower than in placebo	Frequency of high parasite densities lower than in placebo
Figure 1f: Sensitivity of case definition, by parasite density	Same as placebo	At any given density, reduced relative to placebo	At any given density, reduced relative to placebo
Figure 1g: Specificity of case definition by parasite density	Same as placebo	At any given density, increased relative to placebo	Same as placebo
Figure 1h: Efficacy estimate by parasite density cut-off (*x*)	Estimated efficacy increases with cut-off approximates the true efficacy at high cut-off values	Estimated efficacy increase with cut-off and exceeds the true efficacy at high cut-off values	Estimated efficacy increase with cut-off and approximates the true efficacy at high cut-off values
Figure 1i: Power of study, by parasite density cut-off	Reaches a maximum of about 67% at a cut-off of about 10,000/μl	Reaches a maximum of about 87% at a cut-off of about 40,000/μl	Increases to 100% at a parasite density of about 60,000/μl
Estimated efficacy using latent class model	46.1% (18.7%)	55.6% (23.1%)	55.2% (16.5%)
Power using latent class model (1-β)	59.2%	82.4%	71.2%

Similarly, and as a direct consequence of the curves shown in Figure 1c, for these vaccines A and C, but not for vaccine B, the proportion of disease cases attributable to malaria at any given density is less in the active than in the placebo arm (Figure 1d).

The relative frequencies of malaria cases at different parasite densities (Figure 1e) show similar patterns to those of the relative frequencies of all disease cases (Figure 1d), but instead of intersecting at a non-zero point on the vertical axis, the plots pass through the origin, since clinical malaria cannot occur in the absence of parasites.

Integration of the curves in Figure 1e (Equation 3) then gives the curves for the sensitivity of parasite density cut-offs. There are clear differences between the vaccines. At any parasite density the sensitivity for vaccine A is equivalent to that for placebo, but for vaccines B and C it falls below that of placebo. The specificity, in contrast, is the same as placebo for vaccines A and C, but is higher than placebo in vaccine B (Figure 1g).

These differences in sensitivity and specificity have effects on the estimation of efficacy. At high values of *x*, corresponding to high specificity, the mean efficacy estimate (of the 500 simulated trials) for vaccines A and C approaches the true efficacy, while for vaccine B (where specificity in the vaccine arm is higher than in the placebo arm) the efficacy is overestimated. The proportion of trials giving statistically significant results (Figure 1i) (assuming them to have been analysed using a fixed cut-off) gives the power of the study. The power of the trials of vaccines A and C showed maxima at relatively low cut-offs, indicating that different cut-offs must be used if the aim is to avoid bias in the estimate of efficacy, from those used to optimise power.

## Discussion

Field trials of interventions against malaria need to have easily interpretable primary outcome measures in order to make an impact on regulatory and policy decisions. At the same time, field trials represent the main opportunity for experimental study of immuno-epidemiology of malaria and need to be fully exploited to further understanding of the mechanisms of action of the interventions. The analyses demonstrated in this paper are intended to contribute to plans for such secondary analyses.

The three hypothetical vaccines simulated in this study represent limiting cases of the effects of different interventions on clinical malaria. They do not correspond on a one-to-one basis to real vaccines, but rather to possible intervention effects. Any real intervention might have secondary effects on the other measures in addition to a primary effect on force of infection, asexual parasite growth, or on pyrogenic thresholds. Analyses of trial datasets should aim to identify contributions of an intervention to each of these dimensions of protective efficacy.

The primary outcome of most trials is likely to use a single parasite density cut-off that is chosen to give a high specificity in order to reduce underestimation of efficacy since decisions to develop a vaccine depend on the magnitude of protection. However a highly specific case definition does not necessarily result in optimization of study power (Figure 1i) and in early stages of vaccine development it might be most important to to test whether there is any effect at all so a lower cut-off would be more appropriate. There is no reason why a threshold chosen to reduce bias in efficacy should be particularly appropriate for any other purpose and in particular a diagnostic threshold optimized for use in a trial is not necessarily appropriate as a tool in clinical management [12].

Analyses of trial data using such parasitaemia cut-offs have generally not quantified the bias that remains. The true efficacy is defined as *E *= 1 - *I*_*V*_/*I*_*P *_where *I*_*V *_is the case incidence in vaccinees and *I*_*P *_is the case incidence in the placebo arm, and the usual estimate of efficacy is E˜
 MathType@MTEF@5@5@+=feaafiart1ev1aaatCvAUfKttLearuWrP9MDH5MBPbIqV92AaeXatLxBI9gBaebbnrfifHhDYfgasaacH8akY=wiFfYdH8Gipec8Eeeu0xXdbba9frFj0=OqFfea0dXdd9vqai=hGuQ8kuc9pgc9s8qqaq=dirpe0xb9q8qiLsFr0=vr0=vr0dc8meaabaqaciaacaGaaeqabaqabeGadaaakeaacuWGfbqrgaacaaaa@2DCE@ = 1 - *I*_*V*_(*x*)/*I*_*P*_(*x*) where *I*_*V*_(*x*) is the incidence of cases at or above cut-off (x) in the vaccine arm, and *I*_*P*_(*x*) the corresponding incidence in the placebo arm. Assuming the specificity (ψ) of the diagnostic cut-off to be the same in both arms then an estimate of E adjusted for the effects of the imperfect case definition is:

E^=1−IV(x)−(1−ϕ)(1−λ)IP(0)IP(x)−(1−ϕ)(1−λ)IP(0)
 MathType@MTEF@5@5@+=feaafiart1ev1aaatCvAUfKttLearuWrP9MDH5MBPbIqV92AaeXatLxBI9gBaebbnrfifHhDYfgasaacH8akY=wiFfYdH8Gipec8Eeeu0xXdbba9frFj0=OqFfea0dXdd9vqai=hGuQ8kuc9pgc9s8qqaq=dirpe0xb9q8qiLsFr0=vr0=vr0dc8meaabaqaciaacaGaaeqabaqabeGadaaakeaacuWGfbqrgaqcaiabg2da9iabigdaXiabgkHiTmaalaaabaGaemysaK0aaSbaaSqaaiabdAfawbqabaGccqGGOaakcqWG4baEcqGGPaqkcqGHsislcqGGOaakcqaIXaqmcqGHsisliiGacqWFvpGAcqGGPaqkcqGGOaakcqaIXaqmcqGHsislcqWF7oaBcqGGPaqkcqWGjbqsdaWgaaWcbaGaemiuaafabeaakiabcIcaOiabicdaWiabcMcaPaqaaiabdMeajnaaBaaaleaacqWGqbauaeqaaOGaeiikaGIaemiEaGNaeiykaKIaeyOeI0IaeiikaGIaeGymaeJaeyOeI0Iae8x1dOMaeiykaKIaeiikaGIaeGymaeJaeyOeI0Iae83UdWMaeiykaKIaemysaK0aaSbaaSqaaiabdcfaqbqabaGccqGGOaakcqaIWaamcqGGPaqkaaaaaa@5D5A@

where *λ *is the attributable fraction in the placebo arm. A potential improvement in efficacy estimates is to thus to estimate E^
 MathType@MTEF@5@5@+=feaafiart1ev1aaatCvAUfKttLearuWrP9MDH5MBPbIqV92AaeXatLxBI9gBaebbnrfifHhDYfgasaacH8akY=wiFfYdH8Gipec8Eeeu0xXdbba9frFj0=OqFfea0dXdd9vqai=hGuQ8kuc9pgc9s8qqaq=dirpe0xb9q8qiLsFr0=vr0=vr0dc8meaabaqaciaacaGaaeqabaqabeGadaaakeaacuWGfbqrgaqcaaaa@2DCF@ from E˜
 MathType@MTEF@5@5@+=feaafiart1ev1aaatCvAUfKttLearuWrP9MDH5MBPbIqV92AaeXatLxBI9gBaebbnrfifHhDYfgasaacH8akY=wiFfYdH8Gipec8Eeeu0xXdbba9frFj0=OqFfea0dXdd9vqai=hGuQ8kuc9pgc9s8qqaq=dirpe0xb9q8qiLsFr0=vr0=vr0dc8meaabaqaciaacaGaaeqabaqabeGadaaakeaacuWGfbqrgaacaaaa@2DCE@, *λ*, and ψ and to use E^
 MathType@MTEF@5@5@+=feaafiart1ev1aaatCvAUfKttLearuWrP9MDH5MBPbIqV92AaeXatLxBI9gBaebbnrfifHhDYfgasaacH8akY=wiFfYdH8Gipec8Eeeu0xXdbba9frFj0=OqFfea0dXdd9vqai=hGuQ8kuc9pgc9s8qqaq=dirpe0xb9q8qiLsFr0=vr0=vr0dc8meaabaqaciaacaGaaeqabaqabeGadaaakeaacuWGfbqrgaqcaaaa@2DCF@ as an estimate of *E*. If ψ is sufficiently close to unity, then the difference between these two estimates is small.

Exploratory analyses of the behaviour of E^
 MathType@MTEF@5@5@+=feaafiart1ev1aaatCvAUfKttLearuWrP9MDH5MBPbIqV92AaeXatLxBI9gBaebbnrfifHhDYfgasaacH8akY=wiFfYdH8Gipec8Eeeu0xXdbba9frFj0=OqFfea0dXdd9vqai=hGuQ8kuc9pgc9s8qqaq=dirpe0xb9q8qiLsFr0=vr0=vr0dc8meaabaqaciaacaGaaeqabaqabeGadaaakeaacuWGfbqrgaqcaaaa@2DCF@ suggest that it can be sensitive to *x *(Aponte, pers. comm), though it should not be so if the assumptions underlying its estimation are correct. The non-linear logistic regression model most widely used for defining the parasitaemia cut-off[1] assumes a specific parametric form for the relationship between relative risk and parasite density. This can lead to severely biased estimates of the specificity of the cut-off if the relationship happens not to conform to this pattern [10]. This assumption is avoided in the latent class models that we have used in this paper which fit non-parametric curves for this relationship.

It is evident from Figure 1h though that the bias in efficacy does not only arise from lack of specificity in cut-off, and need not always be in the direction of underestimating efficacy. Bias also arises because of the specificity of cut-offs can differ between vaccine and placebo. Our model indicates that this is particularly a problem for asexual blood stage vaccines (vaccine B) (Figure 1g). This leads to the idea that perhaps different cut-offs should be used for vaccine and for placebo groups[7]. To justify this in practice though, it would be necessary to demonstrate a statistically significant difference between trial arms in the specificity vs cut-off relationship. This would be a difficult statistical exercise, (because the specificity is estimated only indirectly), and would lead to considerable difficulties in describing the results convincingly especially if the efficacy proved highly sensitive to the choices of cut-offs in the different groups. Because sample size is determined in order to give adequate power to measure the primary outcome (effect on case incidence), most trials are too small to conclusively demonstrate whether the specificity vs cut-off relationship varies between arms. The decision of the WHO Study Group on Measures of Malaria Vaccine Efficacy not to recommend trial-arm specific cut-offs [9] is therefore probably well-founded.

The most satisfactory alternative to using a single cut-off would probably be to estimate the total number of clinical malaria cases in each arm of the trial by assigning a probability to each fever case, rather than classifying each case dichotomously as above, or below, cut-off. This approach has not so far been used in analyses of clinical trials though it has been proposed as an alternative to the arbitrary choice of a cut-off [12]. It has been used in observational epidemiological studies [13,14]. The preferred estimation method is to use a Bayesian latent class model to estimate the probabilities [11] carrying out this analysis separately for both placebo and vaccine arms. The simulations of this approach presented in Table 2 suggest that it has comparable power to that of the cut-off method. Moreover, interval estimates for all the quantities involved are readily available using software written in Winbugs [15] available from the author.

Such secondary analyses using latent class models, or considering the whole range of possible parasite density cut-offs will also help to identify possible biases in efficacy estimates made using single case definitions, at the same time as analysing the kind of protection. Where multi-centre trials give heterogeneous efficacy estimates, it will be important to examine whether this can be accounted for by differential bias in the primary outcome measurements.

In a real trial the reduction in proportion of individuals infected varies over the trial period, depending on the time course of incidence, patterns of treatment with anti-malarial drugs, and on the variation between individuals in exposure to vectors and responses to vaccination. These factors significantly complicate the analysis of relationships between infection and morbidity because, strictly speaking, the comparison should always be between contemporaneous data. This problem is particularly acute if parasites are cleared at the start of the trial, leading to complicated dynamics of infection and disease during the trial follow-up period. The present simulations do not address the implications of sub-patent parasitaemia. This especially complicates analysis of effects of asexual blood stage vaccines because reduction of parasite densities differentially inflates the proportion of false-negative blood slides in vaccinated individuals.

It follows that the analyses illustrated in this paper represent considerable simplifications of those that might be carried out in a real trial, where these complicating factors need to be taken into account. Nevertheless, when feasible, it would be logical to carry out secondary analyses corresponding to the different panels in Figure 1. Such analyses would help to clarify whether the effect of an intervention corresponds to that anticipated on the basis of the parasite stage that is targeted, and would highlight whether the primary measure of efficacy results from any unexpected behaviour in the parasitological and clinical data used to estimate it.
